# A *de novo* Assembly of the Common Frog (*Rana temporaria*) Transcriptome and Comparison of Transcription Following Exposure to *Ranavirus* and *Batrachochytrium dendrobatidis*

**DOI:** 10.1371/journal.pone.0130500

**Published:** 2015-06-25

**Authors:** Stephen J. Price, Trenton W. J. Garner, Francois Balloux, Chris Ruis, Konrad H. Paszkiewicz, Karen Moore, Amber G. F. Griffiths

**Affiliations:** 1 UCL Genetics Institute, University College London, Darwin Building, Gower Street, London, United Kingdom; 2 Institute of Zoology, Zoological Society of London, London, United Kingdom; 3 Wellcome Trust Biomedical Informatics Hub, Biosciences, Geoffrey Pope Building, University of Exeter, Streatham Campus, Exeter, United Kingdom; 4 Environment and Sustainability Institute, University of Exeter, Penryn Campus, Penryn, Cornwall, United Kingdom; University of South Dakota, UNITED STATES

## Abstract

Amphibians are experiencing global declines and extinctions, with infectious diseases representing a major factor. In this study we examined the transcriptional response of metamorphic hosts (common frog, *Rana temporaria*) to the two most important amphibian pathogens: *Batrachochytrium dendrobatidis* (*Bd*) and *Ranavirus*. We found strong up-regulation of a gene involved in the adaptive immune response (AP4S1) at four days post-exposure to both pathogens. We detected a significant transcriptional response to *Bd*, covering the immune response (innate and adaptive immunity, complement activation, and general inflammatory responses), but relatively little transcriptional response to *Ranavirus*. This may reflect the higher mortality rates found in wild common frogs infected with *Ranavirus *as opposed to *Bd*. These data provide a valuable genomic resource for the amphibians, contribute insight into gene expression changes after pathogen exposure, and suggest potential candidate genes for future host-pathogen research.

## Introduction

Amphibians are currently undergoing a mass extinction event [1]. Two key pathogens are known to be contributing to amphibian population declines and species extinctions: the fungus *Batrachochytrium dendrobatidis* (*Bd*) which causes chytridiomycosis, and the *Iridoviridae* genus *Ranavirus* [2–5]. *Bd* is a non-hyphal zoosporic fungus which causes mortalities on every continent except Antarctica (http://www.bd-maps.net/) and is thought to have caused multiple species extinctions [6]. *Ranaviruses* are large double-stranded DNA viruses, capable of crossing poikilothermic class boundaries, and implicated in mass die-off events and population declines [2,5,7]. *Ranavirus* and *Bd* are both noted for their virulence across a broad range of hosts but previous research on wild and captive animals points to contrasting levels of pathogenicity in European common frogs (*Rana temporaria*). Common frogs are highly susceptible to *Ranavirus* infection in the UK [5,8] and Spain [7] but seem relatively resistant to *Bd* [9]. It is unknown whether this difference in susceptibility reflects differences in the host’s immune response to each pathogen. *De novo* RNAseq offers an ideal opportunity to further our understanding of the host response to *Bd* and *Ranavirus*, allowing the identification of genes and pathways involved in the response to infection.

*Bd* is thought to kill its host by disrupting the cutaneous integrity and function of amphibian skin [10]. Transcriptome sequencing has suggested that more resistant hosts may increase expression of genes involved in skin structure [11] and dramatic up and down-regulation of pathways relating to collagen, fibrinogen, elastin and keratin have been reported in the skin of adult amphibians experimentally infected with *Bd* [12]. Enrichment of inflammatory responses when challenged with *Bd* may be a general response regardless of susceptibility [11,13]. Increased expression of microbial peptides in response to *Bd* infection has also been identified in several species (*Rana sierrae*, *Rana muscosa* adults [12]; *Xenopus tropicalis* adults [14]), indicating that innate immunity is a component of host defence. Robust adaptive immunogenetic responses to *Bd* infection have in general not been observed, but components of innate and adaptive immunity have been shown to operate even in species that are highly susceptible [13]. In addition significant up and down regulation of adaptive immune genes (including the Major Histocompatibility Complex (MHC) class I and II in particular) have been shown in experimentally infected adult ranids [12,14] and a comparison of responses to *Bd* in hosts of varying susceptibility suggested that the ability to escape immunosuppression by mounting T-cell mediated responses may determine resistance [11]. Our understanding of the response of juveniles to *Bd* remains limited.

To date, there has been one study of the transcriptional response of amphibians to *Ranavirus*. *Ambystoma mexicanum* (axolotl) showed a significant immunological response when experimentally challenged with the *Ranavirus Ambystoma tigrinum virus* (ATV) [15]. Wild ambystomatid salamanders are highly susceptible to ATV, however experimental animals appeared to mount an immune response to infection within 24 hours of exposure. Using spleen tissues processed through microarrays, the authors demonstrated changes in the expression of innate immunity genes and the transcriptional response increased through time [15]. Experimental infection of adult *Xenopus laevis* with a strain of *Ranavirus* (FV3) has demonstrated increased expression of pro-inflammatory cytokines e.g. tumour necrosis factor alpha (TNF-α) and interleukin-1β (IL-1β), indicating that (like for *Bd*) the innate immune response is activated following infection [16]. Tadpoles show weaker and more delayed up-regulation of these genes [17]. Specific MHC class Ia gene supertypes have been found to be associated with infection status of adult wild common frog (*R*. *temporaria*) populations, and diseased populations are characterized by more similar supertype frequencies (lower *F*_ST_) than infected populations, indicating pathogen-driven selection on the MHC [18]. This implies that an adaptive immune response to *Ranavirus* occurs in *R*. *temporaria*, and may be important for survival after infection. While adult *Xenopus laevis* are able to clear FV3 infections, tadpoles do not appear to mount an adaptive immune response to FV3, and succumb to infection within a month of inoculation [17]. Again, little is known about the immune response of juveniles. In general, metamorphosis is a critical point in amphibian immunity–the adaptive immune response appears to be limited pre-metamorphosis, the innate immune response is transformed at metamorphosis, and during metamorphosis individuals are thought to experience temporary immunosuppression [19].

In order to better understand the host response to *Bd* and *Ranavirus*, in this study we (i) used RNAseq to generate an annotated de-novo transcriptome for the common frog (*R*. *temporaria*) (ii) conducted comparative expression profiling of the early responses of metamorphic frogs exposed to *Bd* or *Ranavirus* relative to control animals, and (iii) identified candidate genes for future studies on the population-level impacts of these pathogens.

## Methods

### Experimental treatments

*Ranavirus* (RUK13 isolate; [20]) was cultured at 24ºC in Fathead Minnow cells (FHM, Epithelial-like cells from the posterior anal tissue, obtained from the European Collection of Cell Cultures catalogue number 88102401) in maintenance media (EMEM + 10% FBS + 1% L-Glutamine) and quantified on 96 well flat-bottomed cell culture plates using the TCID_50_ method [21]. *Bd* inoculum (Isolate IA 042, *Bd*GPL; [22]) was prepared by culture in mTGhL medium at 18ºC for four days before counting zoospores using a haemocytometer.

*R*. *temporaria* eggs were obtained from a private garden pond in Chessington in the UK with the permission of the landowner [5]. This site has a known history of *Ranavirus* infection, but an unknown history of *Bd* infection. The eggs were hatched and reared through metamorphosis under controlled conditions, showing good survival and no signs of infection. Metamorphs (*n* = 45) were moved into an experimental room (18-21ºC, 33–46% humidity, full spectrum UV light) for acclimatisation one week prior to exposure, and were placed in individual boxes and fed on crickets ad libitum.

This work was carried out under Home Office license (Project Licence numbers PPL 80/2214 and PPL 80/2466) and was approved by the Institute of Zoology Ethics Committee and the University of Exeter Ethical Review Board. In total 45 frogs were used—15 animals for each of three exposure treatments (*Ranavirus*, *Bd*, Control). Animals were checked, cleaned and given fresh water frequently whilst being mindful of causing them distress through unnecessary handling. Exposure was performed in individual tubes with 29ml aged tap water with 1ml of inoculum (*Ranavirus* at 1k TCID_50_/ml or *Bd* at 100k active zoospores/ml), or 30ml tap water for the control treatment, for four hours. Following exposure, the frogs were returned to their individual boxes and examined daily for signs of *Ranavirus* infection (oedema of the eye, skin ulceration, bleeding), which would have served as an endpoint for the experiment but none were observed. Frogs were euthanized according to Schedule 1 to the Animals (Scientific Procedures) Act 1986 four days post-exposure by immersion in fresh 5g/L Tricaine methane sulphonate (MS222, Pharmaq Ltd.) solution neutralized with sodium bicarbonate in accordance with Universities Federation for Animal Welfare guidance to ameliorate suffering. We sampled livers because they are large, easily targeted organs which are important for immunity and are a target organ in ranavirus disease [23]. Livers were immediately dissected, preserved individually in RNAlater solution (Sigma Aldrich), and stored at -80ºC.

### Sequencing, de novo assembly and abundance estimation

Total RNA was extracted from 5–20μg of liver tissue using the Qiagen RNeasy Mini Kit (Qiagen, Valencia USA) using the standard protocol. Each treatment consisted of 15 frogs; three pools were sequenced per treatment, with five individuals per pool. We therefore prepared nine samples for sequencing; 3 replicates per treatment, each consisting of pooled RNA extracted from the livers of 5 individuals. RNA concentration was estimated using a NanoDrop (NanoDrop, Wilmington USA), and concentrations of individual sample extractions were equalized within pools, resulting in a total pool volume of 80μl (concentrations varied between pools, Supporting Information S1 table). The sample concentrations were not normalized. DNase treatment, library preparation and 100 base pair paired-end sequencing were performed at the Wellcome Trust Biomedical Informatics Hub using an Illumina HiSeq 2500 with two samples per lane.

Raw reads were processed with the fastq-mcf package (ea-utils; https://code.google.com/p/ea-utils/wiki/FastqMcf; [24]) to trim low quality bases and adaptor sequences from the ends of reads and remove short reads as well as those containing non-assigned bases (Ns). The following settings were used: quality threshold of 20, minimum remaining sequence length of 35, minimum identity between adapter sequence and clipped sequence of 85%, no Ns permitted, and a minimum clip length of 3. The results were then evaluated using FastQC [25]. Reads from all samples were combined and processed through the standard Trinity pipeline (r2013-02-25 release) for *de novo* RNAseq assembly [26]. Assembly computation requirements were reduced by performing *in silico* normalization on the reads to reduce the total number of reads and remove errors whilst maintaining transcriptome complexity (maximum coverage for reads = 30; minimum kmer coverage for catalogue construction = 2). Isotigs were assembled along Trinity’s three-stage protocol (Inchworm, Chrysalis, Butterfly) with default settings including a minimum isotig length of 200bp. Transcript abundance estimates for each sample were obtained using RSEM (packaged with Trinity) [27].

### Functional annotation and comparative transcription rates

The assembled transcriptome was annotated using Trinotate, a suite of programs for functional annotation of transcriptomes that is suitable for use with non-model organisms. Sequences were processed through a pipeline consisting of a homology search (NCBI-BLAST), protein domain identification (HMMER/PFAM), protein signal prediction (singalP/tmHMM), and comparison to EMBL Uniprot eggNOG and the GO Pathways annotation databases [28–37].

The reference assembly was filtered prior to differential expression analysis so that the assembly used for downstream analyses might better reflect transcribed genes and to reduce the number of comparisons undertaken. Filtering was based on the number of mapped reads. Assembled isotigs were retained only if the FPKM (expected fragments per kilobase of transcript per million fragments sequenced [38]) was greater than or equal to one for all three replicates within one or more treatments (FPKM ≥ 1).

We used the CEGMA pipeline to accurately annotate Core Eukaryotic Genes (CEGs; [39]). CEGMA can be used to assess assembly completeness and the impact of downstream filtering steps via the proportion of core genes present. This proportion is calculated relative to a reference set containing 248 of the most highly conserved CEGs and analysis suggests it is a good metric to assess completeness of draft assemblies [40]. We ran CEGMA with default settings on our reference (unfiltered) and FPKM ≥ 1 filtered assemblies to obtain the number of complete [more than 70% of the protein length aligned to isotig(s)] and partial [alignment length is less than 70% but remains higher than a minimum alignment threshold] core genes that these assemblies contained.

Differential expression analysis was performed with edgeR using a Trinity packaged Perl script. Subsets of differentially expressed transcripts were compiled based on log-fold change of one and Benjamini–Hochberg adjusted p-values [41] to control for false discovery rate [FDR<0.05 to obtain the transcript list and FDR<0.10 for downstream Gene Ontology (GO) term enrichment analysis]. All pairwise comparisons were considered; *Bd* vs. control, *Ranavirus* vs. control, and *Bd* vs. *Ranavirus*. Differentially expressed transcripts in the *Bd* vs. *Ranavirus* comparison were allocated to either *Bd* or *Ranavirus* (S1 Text).

### Analysis of Gene Ontology term enrichment

Bingo (a Cytoscape plugin; [42]) was used to search for GO term enrichment (identifying which GO terms are over or under-represented). Bingo compares the list of differentially expressed products to a user generated list of all genes/transcripts and is therefore of particular use for research on non-model organisms. EdgeR subsets of differentially expressed transcripts at FDR = 0.05 and FDR = 0.10 were analyzed.

## Results

### Sequencing, Assembly and annotation

A total of 1.29 x 10^9^ reads were generated across all nine samples with a mean quality score of 35.2. All raw data is available through EMBL-EBI Array Express, accession number E-MTAB-3632. In total, 199,602 isotigs were generated with an N50 isotig length of 1,086 base pairs and 30,931 isotigs longer than 1,000bp. There were 134,080,068 bases contained in all isotigs with a GC content of 44%. Assembly summary statistics are shown in Table 1. Trinotate generated a list of 48,263 annotated isotigs from 32,486 disconnected subgraphs. These isotigs hit 17,631 genes when orthologs were included and 11,851 genes when excluding orthologs. The species with the highest number of hits were humans (5,605 genes), mouse (3,800), *Xenopus laevis* (1,720), rat (1,382), cow (1,154) and *Xenopus tropicalis* (1,035). In total, 71–76% of reads mapped back to the reference assembly from each set of sample reads (i.e. as part of RSEM analysis); control 73–76%, *Ranavirus 7*1–74%, *Bd* 72–74%.

**Table 1:** Trinity assembly summary statistics.

**Isotig lengths:**	
Minimum isotig length:	201
Maximum isotig length:	18,036
Mean isotig length:	672
Standard deviation of isotig length:	944
Median isotig length:	352
N50 isotig length:	1,086
**Numbers of isotigs:**	
Number of isotigs:	199,602
Number of isotigs more than 1kb in length:	30,931
Number of isotigs in N50:	28,238
**Number of bases in assembled isotigs:**	
Number of bases in all isotigs:	134,080,068
Number of bases in isotigs > = 1kb in length:	69,845,219
GC Content of isotigs:	43.95%

The reference assembly was almost complete when compared to CEGMA’s 248 CEG set, giving us confidence that our methods have yielded an assembly that is a good approximation to the actual transcriptome. In total, 240 (of 248; 97%) complete CEGs and an additional six partial CEGs (total = 246 of 248; 99%) were found in our reference assembly. Summary statistics measuring the completeness of the assembly (broken down by KOG group) are included in S2 Table.

Some genes were lost through our filtering operation but a large majority of CEGs remained. The FPKM ≥ 1 filtered assembly contained 215 (87%) complete genes out of the 248 most highly conserved CEGs. A further five partial genes were present giving a total of 220 CEGs (89%; S2 Table).

### Differential expression

**Fig 1:**
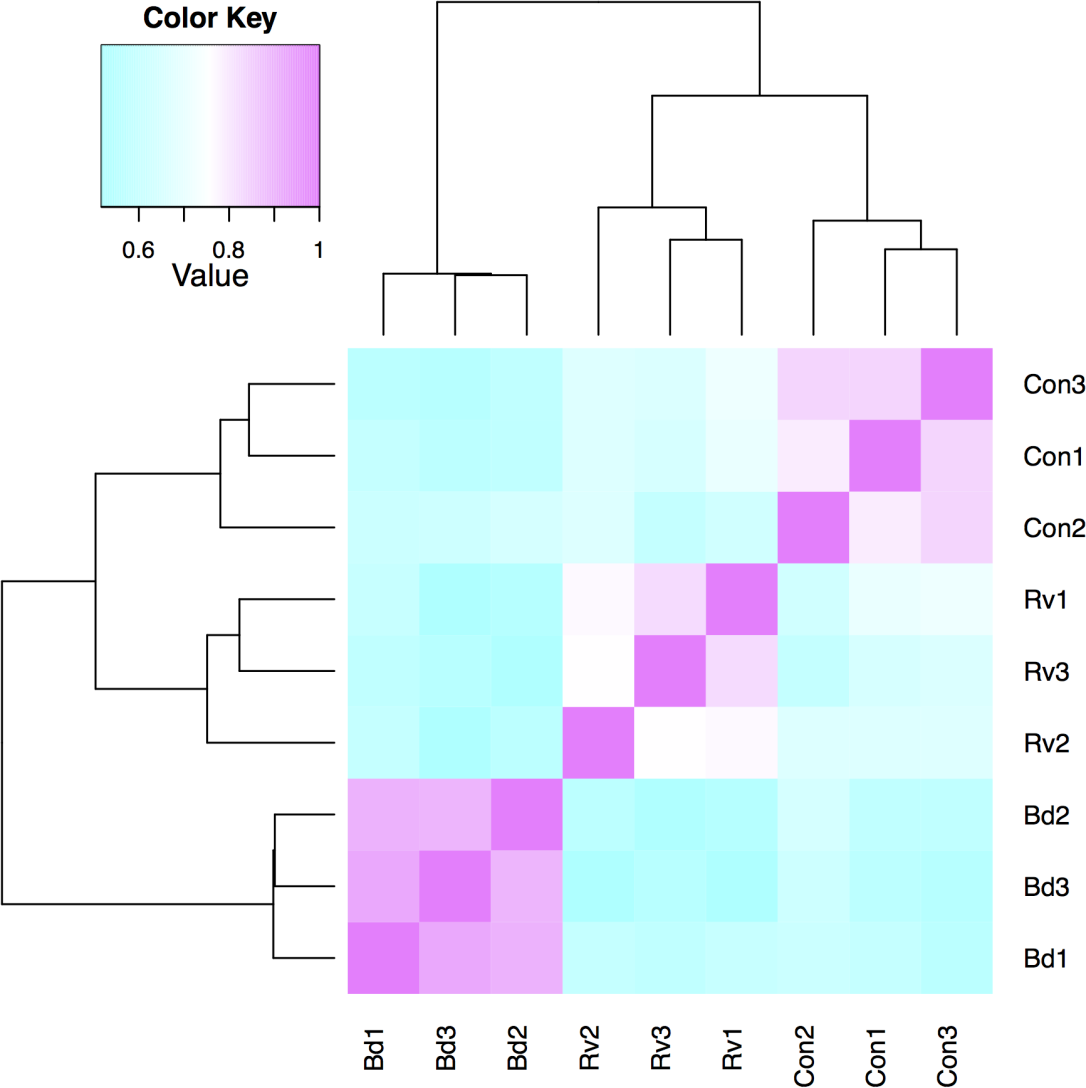
Comparison of transcriptional profiles across all samples. Heatmap visualizing the hierarchically clustered Spearman correlation matrix resulting from a comparison of the transcript expression values (TMM-normalized FPKM) for each pair of samples; Bd = *Batrachochytrium dendrobatidis*, Con = control, Rv = *Ranavirus*.

**Fig 2:**
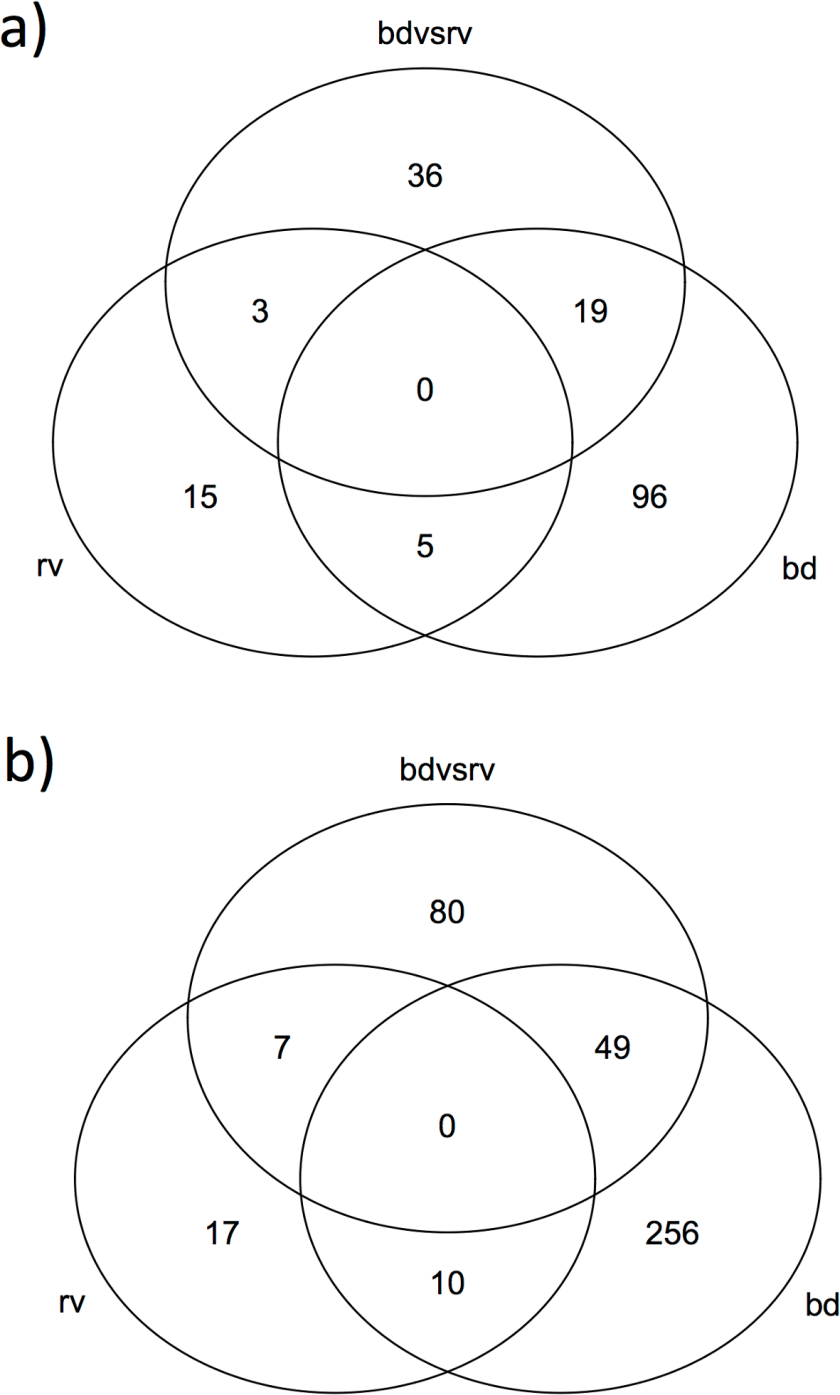
Venn diagrams showing distribution of differentially expressed transcripts across comparisons of treatments. *Bd* vs. control, *Ranavirus* vs. control, and *Bd* vs. *Ranavirus* at a) FDR < 0.05 & b) FDR < 0.10.

Transcriptional profiles of replicates were consistent within treatments, clustering together within treatments when expression values were compared for each pair of samples (Fig 1). Expression values were more similar within the *Ranavirus* and control treatments than in the more divergent *Bd* treatment. The total number of differentially expressed transcripts that we report was affected by the False Discovery Rate (FDR), as well as our procedure for re-allocating some differentially expressed transcripts from the *Bd* vs. *Ranavirus* comparisons to the other comparisons (Table 2; Fig 2). Increasing the FDR increased the number of differentially expressed transcripts in our output. Classifying some of the *Bd* vs. *Ranavirus* transcripts to *Bd* or *Ranavirus* vs. control (S1 Text) also increased the number of transcripts for each pathogen vs. control comparison but reduced the total number of unique differentially expressed transcripts because, for example, some of these transcripts were due to small but opposite effects of the two pathogens compared to controls.

**Table 2:** Summary of the total number of differentially expressed transcripts under alternate FDR regimes: FDR <0.05, FDR<0.10, and differentially expressed transcripts from the *Bd*-*Ranavirus* comparison at FDR<0.10 allocated to one of the other comparisons (S1 Text).

	FDR		
Comparison	<0.05	<0.10	allocated
*Bd* vs. control	120	315	360
*Ranavirus* vs. control	23	34	65
*Bd* vs. *Ranavirus*	58	136	n/a
Both pathogens vs. control	5	10	18
Total unique^†^ transcripts	174	419	407

† some transcripts are differentially expressed in more than one pairwise comparison. This total accounts for this repetition by counting each differentially expressed transcript once only.

**Fig 3:**
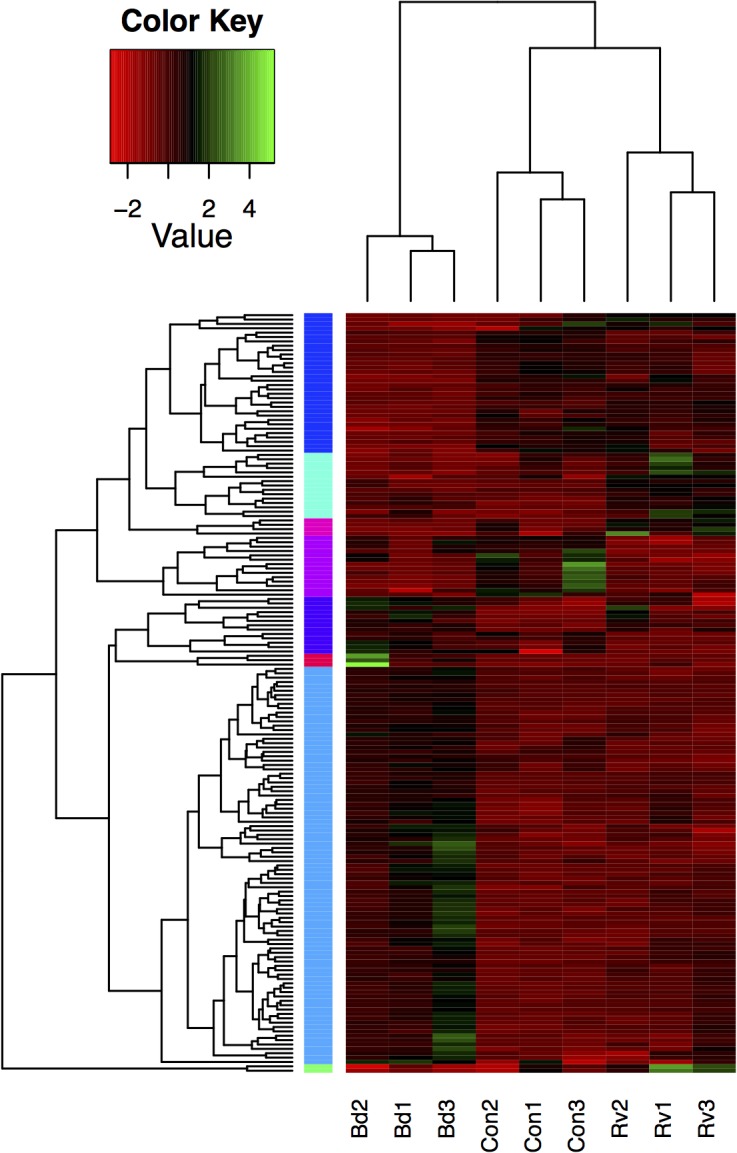
Relative expression of differentially expressed (FDR < 0.05) transcripts (rows) across all samples (columns). Dendrograms show relationships between samples based on expression values (top) and between transcripts based on comparative expression across samples (left). Bd = *Batrachochytrium dendrobatidis*, Con = control, Rv = *Ranavirus*.

**Fig 4:**
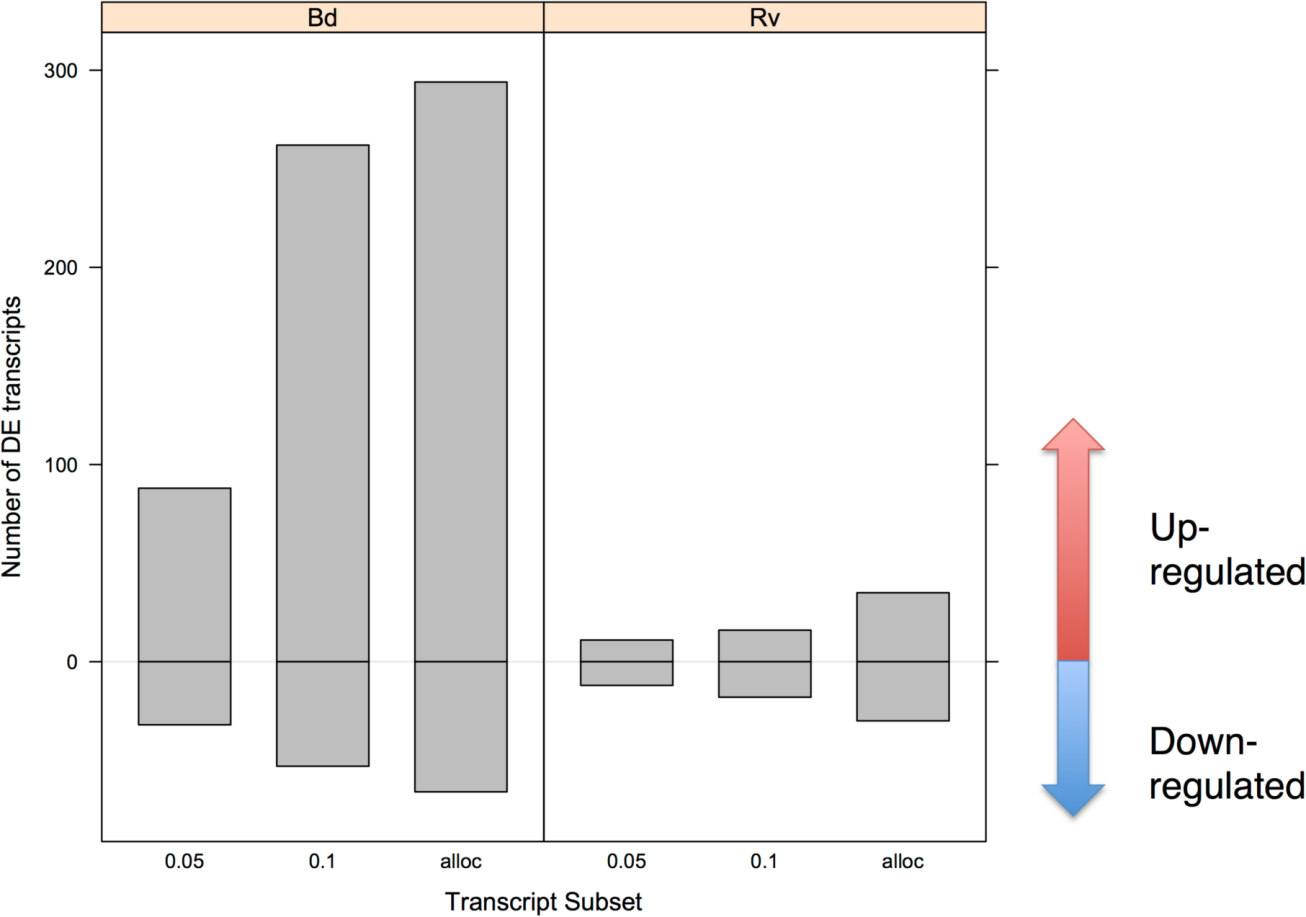
Counts of up- and down-regulated transcripts in response to *Bd* and *Ranavirus*. Data are summarized at alternate false-discovery rate levels (FDR < 0.05 & FDR < 0.10) and following re-allocation of transcripts in the *Bd* vs. *Ranavirus* comparison.

Exposure to *Bd* elicited far greater transcriptional divergence from controls than did exposure to *Ranavirus* (Table 2, Fig 3; 120 differentially expressed transcripts for *Bd* compared to 23 for *Ranavirus* at FDR<0.05). After allocation of the *Bd* vs. *Ranavirus* transcripts to one of the pathogen treatments, there were a total of 360 transcripts (294 up-regulated, 66 down-regulated, 122 annotated) for *Bd* and 65 (35 up-regulated, 30 down-regulated, 16 annotated) for *Ranavirus* (Fig 4). The overall tendency for up-regulated expression in *Bd*-exposed animals was not seen in *Ranavirus*-exposed animals (Fig 4). Amongst annotated transcripts that were differentially expressed after *Bd* challenge, interferon-induced proteins figure prominently through Interferon-stimulated 20kDa exonuclease-like 2 and multiple versions of Interferon-induced very large GTPase 1 proteins (Table 3).

**Table 3:** Differentially expressed transcripts (all comparisons, FDR = 0.05, log-fold-change = 1) that were successfully annotated.

Transcript ID	Comparison	logFC	logCPM	PValue	FDR	Bd1	Bd2	Bd3	Con1	Con2	Con3	Rv1	Rv2	Rv3	Name	Accession	Protein name	Expect Value	Associated with enriched GO term?
comp239933_c4_seq3	bd	10.91	1.33	5.03E-26	9.86E-22	4.41	2.28	2.54	0	0	0	0.4	3.79	0.9	AP4S1_BOVIN	Q3ZBB6	AP-4 complex subunit sigma-1	E:5e-86	
comp229785_c0_seq2	bd	6.08	5.80	3.42E-06	3.20E-03	1.25	112.48	1.45	0.35	0.63	0.76	2.42	1.03	0.5	PRVB_RANES	P02617	Parvalbumin beta	E:2e-55	yes
comp181434_c0_seq2	bd	4.41	2.49	2.27E-06	2.34E-03	1.88	29.12	1.47	0.6	0.5	0.44	0.68	0.51	0.65	ACT3_XENLA	P04752	Actin, alpha skeletal muscle 3	E:0	
comp103973_c0_seq2	bd	4.29	1.64	4.07E-07	6.34E-04	7.53	2.71	14.08	0.43	0.92	0.04	0.37	0.85	0	GVIN1_HUMAN	Q7Z2Y8	Interferon-induced very large GTPase 1	E:2e-61	
comp242668_c0_seq7	bd	3.08	1.02	6.67E-05	2.40E-02	1.98	1.53	1.81	0.35	0	0.34	0.92	0.45	0.21	GVIN1_HUMAN	Q7Z2Y8	Interferon-induced very large GTPase 1	E:6e-88	
comp242668_c0_seq10	bd	2.98	0.30	3.75E-05	1.84E-02	2.72	2.1	2.45	0.1	0.83	0.05	0.41	0.86	0	GVIN1_HUMAN	Q7Z2Y8	Interferon-induced very large GTPase 1	E:3e-117	
comp239447_c0_seq9	bd	2.97	0.98	2.19E-04	3.94E-02	1.8	3.46	1.04	0	0.57	0.29	0.24	1.32	0	ATF4_RAT	Q9ES19	Cyclic AMP-dependent transcription factor ATF-4	E:9e-103	
comp242668_c0_seq9	bd	2.67	0.53	6.01E-06	5.12E-03	3.53	2.87	5.16	0.29	0.46	1.31	2.5	1.01	1.49	GVIN1_HUMAN	Q7Z2Y8	Interferon-induced very large GTPase 1	E:5e-85	
comp91414_c0_seq1	bd	2.56	2.89	8.10E-06	6.62E-03	3.8	24.69	7.71	2.15	2.31	1.96	2.78	1.57	2.08	ALDOA_HUMAN	P04075	Fructose-bisphosphate aldolase A	E:1e-136	
comp224799_c0_seq2	bd	2.56	0.11	1.02E-04	2.90E-02	6.6	2.21	6.65	1.08	1.5	0.3	0.38	1.97	0.41	GVIN1_MOUSE	Q80SU7	Interferon-induced very large GTPase 1	E:7e-06	
comp242668_c0_seq13	bd	2.45	4.11	9.82E-09	2.41E-05	9.74	6.38	10.96	1.29	1.64	2.63	6.22	2.19	3.1	GVIN1_MOUSE	Q80SU7	Interferon-induced very large GTPase 1	E:1e-157	
comp242668_c0_seq6	bd	2.43	2.88	8.95E-08	1.60E-04	5.27	2.75	5.09	0.79	1.23	0.67	1.57	1.49	0.39	GVIN1_HUMAN	Q7Z2Y8	Interferon-induced very large GTPase 1	E:0	
comp242668_c0_seq1	bd	2.42	4.39	4.85E-07	6.34E-04	12.38	7.36	16.85	1.4	3.81	2.5	5.6	4.53	1.79	GVIN1_HUMAN	Q7Z2Y8	Interferon-induced very large GTPase 1	E:3e-68	
comp511272_c0_seq1	bd	2.42	-0.05	1.61E-04	3.29E-02	2.32	2.68	8.25	0.95	1.11	0.71	0.48	1.96	2.44	ANGT_RAT	P01015	Angiotensinogen	E:2e-06	yes
comp242668_c0_seq3	bd	2.37	3.42	4.52E-07	6.34E-04	7.75	4.4	11.14	1.03	1.79	2.3	4.32	2.38	2.82	GVIN1_HUMAN	Q7Z2Y8	Interferon-induced very large GTPase 1	E:0	
comp242026_c1_seq5	bd	2.32	2.31	4.84E-06	4.32E-03	2.18	3.4	3.65	0.98	0.7	0.32	2.49	2.64	1.43	LIAS_XENLA	Q6GQ48	Lipoyl synthase, mitochondrial	E:0	
comp283381_c0_seq1	bd	2.14	1.99	1.33E-04	3.13E-02	9.61	3.37	12.88	2.61	1.42	2.53	4.93	1.48	5.41	AGLUS_METJA	Q58746	Archaeal glutamate synthase [NADPH]	E:6e-154	
comp351995_c0_seq1	bd	2.06	0.65	1.77E-04	3.47E-02	2.61	1.68	4.52	0.91	0.64	0.81	1.4	1.04	1.4	TCPZ_CHICK	Q5ZJ54	T-complex protein 1 subunit zeta	E:7e-112	
comp349197_c0_seq1	bd	2.05	-1.04	2.73E-04	4.58E-02	1.53	1.23	2.2	0.42	0.42	0.46	0.88	0.45	0.7	GCP4_MOUSE	Q9D4F8	Gamma-tubulin complex component 4	E:5e-58	
comp90672_c0_seq1	bd	2.02	-0.55	1.37E-04	3.13E-02	2.37	1.33	2.17	0.53	0.53	0.53	1.02	0.85	1.07	PTN1_CHICK	O13016	Tyrosine-protein phosphatase non-receptor type 1	E:2e-89	
comp225219_c0_seq1	bd	2.01	1.84	1.10E-04	3.05E-02	10.54	6.86	11.4	1.85	4.49	1.52	2.8	3.22	0.24	GVIN1_HUMAN	Q7Z2Y8	Interferon-induced very large GTPase 1	E:1e-36	
comp241555_c2_seq2	bd	1.99	1.50	4.19E-05	1.96E-02	2.79	2.14	1.82	0.36	0.49	1.01	1.93	1.3	0.3	MESH1_XENTR	Q28C98	Guanosine-3',5'-bis(diphosphate) 3'-pyrophosphohydrolase MESH1	E:3e-97	
comp229974_c0_seq1	bd	1.96	2.16	2.46E-06	2.41E-03	4.02	4.63	5.61	1.15	1.37	1.51	1.6	5.18	5.9	UCP2_CYPCA	Q9W725	Mitochondrial uncoupling protein 2	E:1e-171	
comp94070_c0_seq1	bd	1.92	1.66	1.57E-04	3.29E-02	9.58	4.32	11.86	1.91	2.74	2.97	3.68	2.42	4.34	CCNI_HUMAN	Q14094	Cyclin-I	E:1e-73	
comp222706_c0_seq1	bd	1.83	2.97	1.33E-04	3.13E-02	10.14	7.78	22.32	4.03	4.51	4.21	6.39	6.42	7.46	I20L2_BOVIN	Q2YDK1	Interferon-stimulated 20 kDa exonuclease-like 2	E:2e-82	
comp229273_c0_seq1	bd	1.79	1.21	1.36E-04	3.13E-02	1.31	1.18	1.97	0.49	0.49	0.45	0.36	0.46	0.5	RN145_MOUSE	Q5SWK7	RING finger protein 145	E:0	
comp212366_c0_seq1	bd	1.78	3.49	1.39E-04	3.13E-02	20.21	14.26	43.21	7.65	8.68	9.17	11.28	10.4	17.08	SF3A1_BOVIN	A2VDN6	Splicing factor 3A subunit 1	E:5e-51	
comp197838_c0_seq1	bd	1.71	3.96	1.52E-04	3.29E-02	11.96	7.56	22.38	4.25	4.74	5.45	4.97	3.87	9.34	AGFG1_HUMAN	P52594	Arf-GAP domain and FG repeat-containing protein 1	E:3e-174	
comp225693_c0_seq1	bd	1.61	3.51	2.06E-04	3.82E-02	19.41	13.65	33.7	7.4	8.68	8.52	13.25	9.22	9.2	TDX_CYNPY	Q90384	Peroxiredoxin	E:9e-123	
comp241884_c0_seq6	bd	-1.66	2.25	4.48E-05	1.98E-02	0.58	0.6	0.77	2.19	2.2	2.37	1.52	2.12	0.42	METK1_HUMAN	Q00266	S-adenosylmethionine synthase isoform type-1	E:0	
comp242157_c2_seq1	bd	-1.66	2.77	1.60E-04	3.29E-02	0.59	0.68	1.09	2.49	2.03	3.78	1.24	3.31	1.96	DNJB9_MOUSE	Q9QYI6	DnaJ homolog subfamily B member 9	E:3e-115	
comp230044_c0_seq1	bd	-1.78	1.57	2.16E-04	3.92E-02	0.8	1.27	1.39	5.75	3.07	4.02	3.56	2.46	0.94	LDLR1_XENLA	Q99087	Low-density lipoprotein receptor 1	E:2e-149	yes
comp234135_c0_seq1	bd	-1.82	1.79	8.43E-05	2.58E-02	0.89	1.05	1.25	5.47	2.99	3.76	2.91	2.37	1.08	LDLR2_XENLA	Q99088	Low-density lipoprotein receptor 2	E:0	yes
comp234135_c0_seq2	bd	-1.90	1.21	8.52E-05	2.58E-02	1.93	1.43	1.41	8.4	4	6.96	4.33	3.49	1.56	LDLR1_XENLA	Q99087	Low-density lipoprotein receptor 1	E:1e-41	yes
comp225798_c0_seq3	bd	-1.93	3.57	1.12E-05	8.45E-03	6.06	3.65	4.11	26.68	11.04	19.18	15.04	25.74	4.57	GRP78_XENLA	Q91883	78 kDa glucose-regulated protein	E:7e-120	
comp225798_c0_seq1	bd	-2.07	6.00	2.81E-05	1.60E-02	7.83	5.24	12.8	28.98	26.02	68.9	21.59	44.4	21.21	GRP78_XENLA	Q91883	78 kDa glucose-regulated protein	E:0	
comp235980_c0_seq10	bd	-2.34	0.51	1.87E-04	3.60E-02	0.22	0.06	0.33	1.47	0.7	1.31	1.87	1.73	1.16	GPBP1_MOUSE	Q6NXH3	Vasculin	E:8e-146	
comp233358_c0_seq1	bd	-2.71	1.47	8.65E-09	2.41E-05	0.39	0.3	0.29	2.67	2.54	1.83	0.53	0.32	0	ACSM3_MOUSE	Q3UNX5	Acyl-coenzyme A synthetase ACSM3, mitochondrial	E:0	
comp236082_c0_seq2	bd	-2.95	2.10	9.57E-12	3.76E-08	0.33	0.42	0.28	2.7	3.32	2.55	5.57	1.28	1.75	FMO5_RABIT	Q04799	Dimethylaniline monooxygenase [N-oxide-forming] 5	E:0	
comp238162_c2_seq1	bd	-3.34	-0.48	7.85E-08	1.54E-04	0.11	0.02	0.18	1.24	1.05	1.13	0.4	0.5	0.27	OVCA2_XENTR	A4II73	Ovarian cancer-associated gene 2 protein homolog	E:2e-110	
comp239921_c0_seq17	bd	-4.27	2.03	1.46E-05	1.06E-02	0.04	0	0.37	2.45	2.02	4.76	3.95	0.11	1.81	ECH1_RAT	Q62651	Delta(3,5)-Delta(2,4)-dienoyl-CoA isomerase, mitochondrial	E:6e-150	
comp242544_c0_seq7	bd	-6.32	1.26	4.00E-21	3.92E-17	0	0.07	0.04	2.48	3.86	2.68	0.34	4.06	1.03	CK054_XENLA	Q6GME2	Ester hydrolase C11orf54 homolog	E:0	
comp234419_c0_seq2	bd	-7.46	0.76	4.32E-07	6.34E-04	0.01	0	0	0	1.75	1.95	1.16	1.45	2.04	FA73B_XENLA	Q6GR21	Protein FAM73B	E:0	
comp229785_c0_seq2	bd_rv	4.90	5.89	1.42E-04	4.89E-02	1.25	112.48	1.45	0.35	0.63	0.76	2.42	1.03	0.5	PRVB_RANES	P02617	Parvalbumin beta	E:2e-55	yes
comp103973_c0_seq2	bd_rv	4.55	1.67	3.77E-07	5.28E-04	7.53	2.71	14.08	0.43	0.92	0.04	0.37	0.85	0	GVIN1_HUMAN	Q7Z2Y8	Interferon-induced very large GTPase 1	E:2e-61	
comp181434_c0_seq2	bd_rv	4.16	2.56	7.23E-06	5.46E-03	1.88	29.12	1.47	0.6	0.5	0.44	0.68	0.51	0.65	ACT3_XENLA	P04752	Actin, alpha skeletal muscle 3	E:0	
comp224799_c0_seq2	bd_rv	2.67	0.13	1.46E-04	4.94E-02	6.6	2.21	6.65	1.08	1.5	0.3	0.38	1.97	0.41	GVIN1_MOUSE	Q80SU7	Interferon-induced very large GTPase 1	E:7e-06	
comp214878_c0_seq1	bd_rv	2.65	3.17	1.25E-04	4.64E-02	16.34	12.63	9.1	10.8	0.63	0	0.39	1.37	4.6	MFAP4_BOVIN	P55918	Microfibril-associated glycoprotein 4	E:7e-74	
comp91414_c0_seq1	bd_rv	2.56	2.94	2.16E-05	1.32E-02	3.8	24.69	7.71	2.15	2.31	1.96	2.78	1.57	2.08	ALDOA_HUMAN	P04075	Fructose-bisphosphate aldolase A	E:1e-136	
comp229401_c0_seq1	bd_rv	2.14	1.63	2.95E-05	1.70E-02	3.67	2.54	4.79	0.75	0.63	3.05	1.23	0.61	0.92	NB5R3_BOVIN	P07514	NADH-cytochrome b5 reductase 3	E:4e-155	
comp242668_c0_seq6	bd_rv	2.10	2.98	9.08E-05	3.71E-02	5.27	2.75	5.09	0.79	1.23	0.67	1.57	1.49	0.39	GVIN1_HUMAN	Q7Z2Y8	Interferon-induced very large GTPase 1	E:0	
comp215007_c0_seq2	bd_rv	2.09	-0.04	8.85E-05	3.69E-02	2.06	1.91	0.9	1.06	1.57	1.96	0.34	0.59	0.31	ABCA8_HUMAN	O94911	ATP-binding cassette sub-family A member 8	E:1e-87	
comp229273_c0_seq1	bd_rv	1.90	1.23	1.10E-04	4.31E-02	1.31	1.18	1.97	0.49	0.49	0.45	0.36	0.46	0.5	RN145_MOUSE	Q5SWK7	RING finger protein 145	E:0	
comp237274_c0_seq1	bd_rv	1.57	3.80	7.01E-05	3.12E-02	12.22	8.48	9.59	5.89	6.67	5.81	3.96	4.64	2.63	GLCTK_RAT	Q0VGK3	Glycerate kinase	E:5e-166	
comp234903_c0_seq1	bd_rv	-2.16	1.76	1.43E-05	1.00E-02	0.36	0.25	0.54	0.59	3.55	0.95	1.53	2.32	1.89	RBM5_XENTR	A4IGK4	RNA-binding protein 5	E:0	
comp203468_c0_seq1	bd_rv	-2.49	1.52	6.96E-07	8.03E-04	1.47	1.27	0.69	9.75	0	6.96	4.56	8.09	8.14	IGJ_MOUSE	P01592	Immunoglobulin J chain	E:2e-44	yes
comp235980_c0_seq10	bd_rv	-2.78	0.95	5.85E-06	4.59E-03	0.22	0.06	0.33	1.47	0.7	1.31	1.87	1.73	1.16	GPBP1_MOUSE	Q6NXH3	Vasculin	E:8e-146	
comp236082_c0_seq2	bd_rv	-2.95	2.16	6.42E-07	7.87E-04	0.33	0.42	0.28	2.7	3.32	2.55	5.57	1.28	1.75	FMO5_RABIT	Q04799	Dimethylaniline monooxygenase [N-oxide-forming] 5	E:0	
comp239921_c0_seq2	bd_rv	-3.87	2.53	6.42E-05	3.00E-02	0.04	0.95	0.05	1.76	0.98	12.33	9.38	4.76	1.84	ECH1_RAT	Q62651	Delta(3,5)-Delta(2,4)-dienoyl-CoA isomerase, mitochondrial	E:6e-150	
comp242544_c0_seq7	bd_rv	-5.50	0.57	1.97E-08	4.84E-05	0	0.07	0.04	2.48	3.86	2.68	0.34	4.06	1.03	CK054_XENLA	Q6GME2	Ester hydrolase C11orf54 homolog	E:0	
comp234419_c0_seq2	bd_rv	-7.77	1.16	3.74E-23	7.35E-19	0.01	0	0	0	1.75	1.95	1.16	1.45	2.04	FA73B_XENLA	Q6GR21	Protein FAM73B	E:0	
comp239933_c4_seq3	rv	9.74	0.31	1.57E-13	1.03E-09	4.41	2.28	2.54	0	0	0	0.4	3.79	0.9	AP4S1_BOVIN	Q3ZBB6	AP-4 complex subunit sigma-1	E:5e-86	
comp235822_c0_seq8	rv	4.32	2.04	7.02E-16	6.89E-12	0	1.01	0	0.23	0.06	0.16	2.78	3.2	2.94	NB5R3_BOVIN	P07514	NADH-cytochrome b5 reductase 3	E:2e-161	
comp236322_c0_seq1	rv	2.28	3.56	2.71E-08	1.33E-04	1.63	1.33	2.11	1.16	0.88	1.09	4.48	3.56	6.94	POL2_MOUSE	P11369	Retrovirus-related Pol polyprotein LINE-1	E:3e-172	
comp215007_c0_seq2	rv	-1.89	-0.28	3.24E-05	3.09E-02	2.06	1.91	0.9	1.06	1.57	1.96	0.34	0.59	0.31	ABCA8_HUMAN	O94911	ATP-binding cassette sub-family A member 8	E:1e-87	
comp240300_c2_seq5	rv	-1.97	2.66	3.58E-06	6.39E-03	5.84	3.81	2.69	3.15	8.03	7.01	1.41	1.61	1.52	CI064_HUMAN	Q5T6V5	UPF0553 protein C9orf64	E:6e-167	
comp227497_c0_seq1	rv	-2.01	7.16	2.68E-06	5.25E-03	8.5	27.9	43.39	69.64	112.17	154.2	17.51	27.83	35.5	CP7A1_HUMAN	P22680	Cholesterol 7-alpha-monooxygenase	E:0	

. bd = *Bd* vs. control, rv = *Ranavirus* vs. control, bd_rv = *Bd* vs. *Ranavirus*

Five transcripts were significantly differentially expressed in both the *Ranavirus* vs. control and *Bd* vs. control sets (FDR<0.05). Three of these were up-regulated after exposure to both pathogens (including the two with the highest logFC change values of all transcripts in both treatments) and two were down-regulated. The AP-4 complex subunit sigma-1 is the only one of these five transcripts that was successfully annotated, and was associated with the highest logFC increase in both treatments (10.9 and 9.74 fold up-regulation in *Bd*, and *Ranavirus* treatments respectively). At the FDR threshold of p<0.10, ten transcripts were significantly differentially expressed in both the *Bd* vs. control and *Ranavirus* vs. control comparisons. Of these, four were up-regulated in both comparisons and six down-regulated. Four annotations in addition to the AP-4 complex were obtained, including (i) Cholesterol 7-alpha-monooxygenase which may be involved in xenobiotic metabolism, (ii) Acyl-coenzyme A synthetase ACSM3, a mitochondrial gene involved in lipid and/or fatty acid metabolism, (iii) Protein CutA homolog possibly involved in metal ion response, and (iv) Protein FAM136A, another mitochondrial protein. Differentially expressed transcripts with annotation are summarized in Table 3, and the full data is available in S1 File.

### GO term enrichment

Enriched GO terms (Table 4; adjusted P-value<0.05) clustered in cell signalling, immunity, inflammation and metabolism. In total, 103 GO terms were significantly enriched in animals challenged with *Bd* (at 5% level after adjusting for multiple comparisons) compared to the reference set (see Table 4). When parent GO terms were examined, 14 of the 20 differentially expressed transcripts under the “metabolic process” parent GO term (GO:0008152) were up-regulated in *Bd* compared to controls. All transcripts related to “Biological regulation” (GO:0065007) were up-regulated. Immune system processes (GO:0006958), inflammatory responses (GO:0006954) and response to stimulus (GO:0050896) were also generally up-regulated (driven by complement activation) though there was also a down-regulation of Immunoglobulin J chain. No GO terms were enriched in the *Ranavirus* challenged animals, and no GO terms (regardless of treatment) were significantly enriched when differentially expressed transcripts in the FDR < 0.05 lists only were used for GO enrichment analyses.

**Table 4:** Enriched GO terms associated with differentially expressed transcripts in the *Bd* vs. control comparison (adjusted P-value <0.05).

GO term description	adjusted PValue	pValue	GO ID	Total Annotated seqs	Individual GO term total	Total DE set	Individual GO term total in DE set
cellular ketone metabolic process	5.51E-08	1.64E-10	GO:0042180	36661	1876	92	23
organic acid metabolic process	5.51E-08	1.73E-10	GO:0006082	36661	1881	92	23
oxoacid metabolic process	5.51E-08	1.05E-10	GO:0043436	36661	1834	92	23
carboxylic acid metabolic process	5.51E-08	1.05E-10	GO:0019752	36661	1834	92	23
negative regulation of endopeptidase activity	1.04E-05	4.08E-08	GO:0010951	36661	130	92	7
monocarboxylic acid metabolic process	1.11E-05	5.23E-08	GO:0032787	36661	1047	92	15
small molecule metabolic process	5.84E-05	3.21E-07	GO:0044281	36661	5068	92	32
negative regulation of peptidase activity	1.14E-04	7.16E-07	GO:0010466	36661	198	92	7
regulation of lipid biosynthetic process	1.46E-04	1.03E-06	GO:0046890	36661	209	92	7
succinate metabolic process	1.62E-04	1.27E-06	GO:0006105	36661	9	92	3
complement activation, classical pathway	3.95E-04	3.41E-06	GO:0006958	36661	161	92	6
humoral immune response mediated by circulating immunoglobulin	4.03E-04	3.79E-06	GO:0002455	36661	164	92	6
humoral immune response	4.07E-04	4.15E-06	GO:0006959	36661	258	92	7
positive regulation of G-protein coupled receptor protein signaling pathway	5.09E-04	5.59E-06	GO:0045745	36661	46	92	4
positive regulation of lipid storage	8.41E-04	9.90E-06	GO:0010884	36661	53	92	4
regulation of triglyceride biosynthetic process	9.83E-04	1.23E-05	GO:0010866	36661	56	92	4
complement activation, alternative pathway	1.19E-03	1.59E-05	GO:0006957	36661	124	92	5
activation of plasma proteins involved in acute inflammatory response	1.39E-03	2.08E-05	GO:0002541	36661	221	92	6
complement activation	1.39E-03	1.97E-05	GO:0006956	36661	219	92	6
immunoglobulin mediated immune response	2.25E-03	3.53E-05	GO:0016064	36661	243	92	6
B cell mediated immunity	2.29E-03	3.78E-05	GO:0019724	36661	246	92	6
negative regulation of hydrolase activity	2.42E-03	4.32E-05	GO:0051346	36661	508	92	8
regulation of triglyceride metabolic process	2.42E-03	4.37E-05	GO:0090207	36661	77	92	4
fatty acid metabolic process	2.59E-03	5.09E-05	GO:0006631	36661	674	92	9
regulation of endopeptidase activity	2.59E-03	5.09E-05	GO:0052548	36661	381	92	7
regulation of peptidase activity	2.71E-03	5.52E-05	GO:0052547	36661	386	92	7
lipid metabolic process	3.48E-03	7.65E-05	GO:0006629	36661	2370	92	17
lymphocyte mediated immunity	3.48E-03	7.59E-05	GO:0002449	36661	279	92	6
regulation of lipid storage	3.53E-03	8.04E-05	GO:0010883	36661	90	92	4
regulation of activation of membrane attack complex	3.70E-03	9.28E-05	GO:0001969	36661	6	92	2
positive regulation of complement activation	3.70E-03	9.28E-05	GO:0045917	36661	6	92	2
positive regulation of activation of membrane attack complex	3.70E-03	9.28E-05	GO:0001970	36661	6	92	2
regulation of lipid metabolic process	3.83E-03	9.91E-05	GO:0019216	36661	424	92	7
positive regulation of glucose transport	4.54E-03	1.21E-04	GO:0010828	36661	100	92	4
protein maturation by peptide bond cleavage	4.85E-03	1.45E-04	GO:0051605	36661	314	92	6
adaptive immune response based on somatic recombination of immune receptors built from immunoglobulin superfamily domains	4.85E-03	1.37E-04	GO:0002460	36661	311	92	6
protein maturation	4.85E-03	1.35E-04	GO:0051604	36661	446	92	7
cellular respiration	4.85E-03	1.43E-04	GO:0045333	36661	197	92	5
regulation of glucose transport	5.26E-03	1.61E-04	GO:0010827	36661	202	92	5
sterol metabolic process	5.74E-03	1.80E-04	GO:0016125	36661	327	92	6
leukocyte mediated immunity	7.24E-03	2.33E-04	GO:0002443	36661	343	92	6
adaptive immune response	8.24E-03	2.72E-04	GO:0002250	36661	353	92	6
acute inflammatory response	8.60E-03	3.11E-04	GO:0002526	36661	362	92	6
positive regulation of type II hypersensitivity	8.60E-03	3.38E-04	GO:0002894	36661	11	92	2
positive regulation of type IIa hypersensitivity	8.60E-03	3.38E-04	GO:0001798	36661	11	92	2
positive regulation of myeloid leukocyte mediated immunity	8.60E-03	3.38E-04	GO:0002888	36661	11	92	2
regulation of type II hypersensitivity	8.60E-03	3.38E-04	GO:0002892	36661	11	92	2
regulation of type IIa hypersensitivity	8.60E-03	3.38E-04	GO:0001796	36661	11	92	2
activation of immune response	8.60E-03	3.32E-04	GO:0002253	36661	517	92	7
respiratory electron transport chain	8.60E-03	3.09E-04	GO:0022904	36661	52	92	3
steroid metabolic process	1.01E-02	4.03E-04	GO:0008202	36661	534	92	7
translation	1.04E-02	4.26E-04	GO:0006412	36661	539	92	7
generation of precursor metabolites and energy	1.06E-02	4.40E-04	GO:0006091	36661	714	92	8
regulation of G-protein coupled receptor protein signaling pathway	1.06E-02	4.51E-04	GO:0008277	36661	141	92	4
regulation of acute inflammatory response to antigenic stimulus	1.20E-02	5.56E-04	GO:0002864	36661	14	92	2
positive regulation of acute inflammatory response to antigenic stimulus	1.20E-02	5.56E-04	GO:0002866	36661	14	92	2
positive regulation of hypersensitivity	1.20E-02	5.56E-04	GO:0002885	36661	14	92	2
regulation of hypersensitivity	1.20E-02	5.56E-04	GO:0002883	36661	14	92	2
positive regulation of protein amino acid phosphorylation	1.20E-02	5.19E-04	GO:0001934	36661	399	92	6
organic acid biosynthetic process	1.21E-02	5.81E-04	GO:0016053	36661	568	92	7
carboxylic acid biosynthetic process	1.21E-02	5.81E-04	GO:0046394	36661	568	92	7
protein processing	1.45E-02	7.04E-04	GO:0016485	36661	423	92	6
positive regulation of inflammatory response to antigenic stimulus	1.45E-02	7.31E-04	GO:0002863	36661	16	92	2
energy derivation by oxidation of organic compounds	1.45E-02	7.30E-04	GO:0015980	36661	426	92	6
positive regulation of phosphorylation	1.54E-02	7.85E-04	GO:0042327	36661	432	92	6
regulation of inflammatory response to antigenic stimulus	1.79E-02	9.28E-04	GO:0002861	36661	18	92	2
regulation of transport	2.08E-02	1.14E-03	GO:0051049	36661	1705	92	12
cellular lipid metabolic process	2.08E-02	1.12E-03	GO:0044255	36661	1701	92	12
positive regulation of phosphorus metabolic process	2.08E-02	1.16E-03	GO:0010562	36661	466	92	6
positive regulation of phosphate metabolic process	2.08E-02	1.16E-03	GO:0045937	36661	466	92	6
cholesterol metabolic process	2.08E-02	1.11E-03	GO:0008203	36661	309	92	5
positive regulation of protein maturation by peptide bond cleavage	2.24E-02	1.27E-03	GO:0010954	36661	21	92	2
positive regulation of immune response	2.26E-02	1.29E-03	GO:0050778	36661	652	92	7
tricarboxylic acid cycle	2.48E-02	1.44E-03	GO:0006099	36661	88	92	3
electron transport chain	2.51E-02	1.48E-03	GO:0022900	36661	194	92	4
regulation of cellular ketone metabolic process	2.57E-02	1.53E-03	GO:0010565	36661	196	92	4
cellular amino acid biosynthetic process	2.63E-02	1.59E-03	GO:0008652	36661	198	92	4
acetyl-CoA catabolic process	2.67E-02	1.63E-03	GO:0046356	36661	92	92	3
small molecule catabolic process	2.74E-02	1.70E-03	GO:0044282	36661	1314	92	10
negative regulation of catalytic activity	2.80E-02	1.76E-03	GO:0043086	36661	886	92	8
positive regulation of extracellular matrix constituent secretion	3.60E-02	2.51E-03	GO:0003331	36661	1	92	1
regulation of extracellular matrix constituent secretion	3.60E-02	2.51E-03	GO:0003330	36661	1	92	1
physiological cardiac muscle hypertrophy	3.60E-02	2.51E-03	GO:0003301	36661	1	92	1
cell growth involved in cardiac muscle cell development	3.60E-02	2.51E-03	GO:0061049	36661	1	92	1
dicarboxylic acid metabolic process	3.60E-02	2.51E-03	GO:0043648	36661	107	92	3
fibroblast proliferation	3.60E-02	2.51E-03	GO:0048144	36661	1	92	1
coenzyme catabolic process	3.60E-02	2.38E-03	GO:0009109	36661	105	92	3
response to muscle activity involved in regulation of muscle adaptation	3.60E-02	2.51E-03	GO:0014873	36661	1	92	1
aerobic respiration	3.60E-02	2.38E-03	GO:0009060	36661	105	92	3
cellular amino acid metabolic process	3.71E-02	2.62E-03	GO:0006520	36661	739	92	7
regulation of immune response	3.76E-02	2.69E-03	GO:0050776	36661	949	92	8
positive regulation of B cell mediated immunity	3.95E-02	2.94E-03	GO:0002714	36661	32	92	2
positive regulation of immunoglobulin mediated immune response	3.95E-02	2.94E-03	GO:0002891	36661	32	92	2
positive regulation vascular endothelial growth factor production	3.95E-02	2.94E-03	GO:0010575	36661	32	92	2
regulation of vascular endothelial growth factor production	3.95E-02	2.94E-03	GO:0010574	36661	32	92	2
purine ribonucleoside triphosphate metabolic process	4.11E-02	3.09E-03	GO:0009205	36661	567	92	6
positive regulation of acute inflammatory response	4.30E-02	3.32E-03	GO:0002675	36661	34	92	2
purine nucleoside triphosphate metabolic process	4.30E-02	3.34E-03	GO:0009144	36661	576	92	6
oxidation reduction	4.30E-02	3.33E-03	GO:0055114	36661	243	92	4
ribonucleoside triphosphate metabolic process	4.63E-02	3.64E-03	GO:0009199	36661	586	92	6
positive regulation of humoral immune response	4.68E-02	3.71E-03	GO:0002922	36661	36	92	2
cofactor catabolic process	4.76E-02	3.81E-03	GO:0051187	36661	124	92	3
heterocycle metabolic process	4.96E-02	4.01E-03	GO:0046483	36661	1242	92	9

## Discussion

We investigated the response of an anuran host (*R*. *temporaria*) to the fungal pathogen *Bd* and the viral pathogen, *Ranavirus*, and detected a significant transcriptional response to *Bd*. Enriched GO terms involved the major arms of the immune response (innate and adaptive immunity and complement activation) as well as metabolic processes. Elements of the adaptive immune response were significantly differentially expressed in animals exposed to *Bd* and those exposed to *Ranavirus*, and this transcriptional response occurred at only four days post-exposure and before signs of disease consistent with ranavirosis were observed. Despite this, the overall response to *Ranavirus* was extremely limited. Variation between pools within treatments resulted in low power to detect differential expression when accounting for multiple comparisons (False Discovery Rate). However, this conservative approach allows confidence in the results and likely includes the genes with the largest transcriptional changes.

Perhaps the most notable result is the strong up-regulation of elements of the adaptive immunity, in response to either pathogen. The highest log fold-change for both pathogen treatments was successfully annotated to the AP-4 complex subunit sigma-1 gene (AP4S1). This is a subunit of a protein coat that is involved in targeting proteins from the trans-Golgi network to the endosomal-lysosomal system (http://www.uniprot.org/uniprot/Q9Y587). The trans-Golgi network is the location for the loading of cytokines (cytokines modulate the balance between the humoral and cell-based adaptive immune responses) with signal peptides into vesicles or carriers for delivery to the cell surface or other organelles [43]. The endosomal-lysosomal proteolysis system appears to be crucial in the immune system, in particular by binding class II MHC molecules to create ligands for antigen recognition by the T lymphocyte system. This process of antigen processing is important for immunity to pathogens as well as for the identification of self-peptides [44]. This result indicates that AP4S1 may be a particularly important candidate gene for future studies of amphibian host-pathogen biology. To date, we are unaware of any studies focusing on this gene in this context.

It is also notable that Interferon-stimulated 20 kDa exonuclease-like 2 and multiple versions of an Interferon-induced very large GTPase 1 were significantly up-regulated in the *Bd* treatment (Table 2). Interferon-induced very large GTPases are thought to mobilise effectors against a broad range of invading pathogens [45]. This result suggests that we have sampled these frogs after an initial innate response and at the point of mobilising other pathways. The result further suggests that these hosts are mounting a major phagocytic response to *Bd* challenge, an anti-fungal response which has previously been shown to be unimpaired during experimental chytrid infection [46].

We found a much stronger transcriptional response to *Bd* exposure than to *Ranavirus*. Our results broadly reflect what is seen in the wild. *R*. *temporaria* populations in the UK have undergone serious declines in the face of recurrent FV3-like *Ranavirus* die-offs [5] and are involved in multi-host mass mortality events associated with infection with a newly emerging *Ranavirus* lineage in Spain [7]. This host is highly susceptible to *Ranavirus* and it seems likely that infected individuals are failing to mount an effective immune response. In contrast, common frogs are considered relatively resistant to *Bd* [9], which may reflect effective immunity. Our observations of a weak transcriptional response to *Ranavirus* are therefore consistent with the higher pathogenicity of *Ranavirus* relative to *Bd* in this host as well as potential immune evasion of *Ranavirus* (reviewed in [17]).

However, the limited overall response to *Ranavirus* challenge that we detected was surprising in the light of previous work. Cotter *et al*. exposed Ambystomatid salamanders to ATV and used a custom microarray to measure host response in spleens at time-points between 24 hours and 6 days [15]. Ambystomatids are naturally infected with ATV in North America and are highly susceptible [47] but immune response (including phagocytosis, cytokine signalling, and complement activation) was detected as early as 24 hours post exposure and transcriptional response increased through the six day experimental period [15]. Differences in methodological approach and host-pathogen system might explain the contrasting findings. Tissue type, dose and exposure route all varied between studies and the host species investigated are highly divergent. The viruses utilised are also divergent. ATV is closely related to fish *Ranaviruses* and may represent a recent host jump [48]. ATV also seems more specialized for salamanders over other amphibians [49]. On the other hand, FV3-like viruses have been more frequently implicated in multi-host die-offs [2] (and may therefore represent better immune evaders) and are the more derived lineage of amphibian-like ranaviruses.

Previous work has shown increased expression of Immunoglobulin Y and activation-induced cytidine deaminase (AID) after *Ranavirus* (FV3) exposure in *Xenopus laevis*, indicating that B-cells are activated, and antibodies to *Ranavirus* have been detected two to six months post-exposure in this host species [50–52]. As well as this adaptive immune response, there was a rapid up-regulation of pro-inflammatory genes (Arginase 1, IL-1B, TNF-a, largely produced by macrophages), and an increased abundance of macrophages and antimicrobial peptides, which are known to inactivate FV3 *in vitro* [52–54]. Other studies indicate that we might expect to detect changes in MHC class I gene expression under *Ranavirus* infection, however it is clear that MHC expression is age-dependent. Pre-metamorphic *Xenopus* tadpoles do not express MHC class Ia genes, while adults do–in juveniles, the stage used in our experiment, expression is weaker than in adults [55]. We saw evidence for an adaptive immune response being initiated (AP4S1 up-regulation) but little evidence of further responses in our *Ranavirus* vs. control comparison.

Rosenblum *et al*. also reported significant transcriptional changes in Interferon-related genes in *Bd*-exposed animals, although they found many of these were only expressed at 16 days as opposed to 3 days post-exposure [12]. Our data demonstrates that in *R*. *temporaria*, these pathways are already active four days post-exposure. *Bd* may suppress T-cell mediated responses to infection [13] and resistance to *Bd* may be partly due to an ability to overcome this [11]. Here we have demonstrated the enrichment of lymphocyte and leukocyte mediated immunity in *R*. *temporaria*. In addition to immunological responses, Rosenblum *et al*. reported differential expression in a range of skin integrity, cellular stress, and homeostasis genes—the majority of these detected 16 days post-exposure [12]. *Bd* disruption of host skin integrity is a key disease process [10] and resistant hosts may mitigate this effect through increased expression of genes contributing to skin structure [11]. Although we sampled livers and not skin, we also observed this type of structural response to *Bd* through up-regulation of genes involved in fibroblast proliferation as well as up-regulation of actin and Galectin-3.

Previous work has shown that particular MHC class IIB alleles are associated with host resistance to *Bd* across a range of hosts varying in their susceptibility to the pathogen [56]. Rosenblum *et al*. reported down-regulation of MHC class I and II in the spleen and up-regulation of MHC class I and II in the skin in *R*. *mucosa* experimentally infected with *Bd*, however these were predominantly shown after 16 days post-exposure and not after only three days post-exposure [12]. We saw no changes in MHC expression in animals exposed to *Bd*.

A more relaxed false discovery rate threshold is expected to yield an increased number of differentially expressed genes but this effect appeared stronger in our *Bd* treatment. The relatively large number of genes withstanding the less stringent FDR correction (0.1) relative to the more conservative one (0.05) suggests that a large number of genes were differentially expressed between treatments but that the amplitude of this change was only modest. Our experimental design involved sampling animals at an early time-point, four days after exposure and prior to any observed mortality or observed signs of disease. This design has enabled identification of changes due to pathogen exposure rather than merely identifying differences between healthy and dying hosts. Previous experiments have pointed to reduced transcriptional responses at early time points post-exposure (3 days) relative to late time-points (16 days) in *Rana* species exposed to *Bd*, with the majority of transcriptional changes only becoming significant later [12]. Innate immune response to *Ranavirus* in *X*. *laevis* peaks at six days post-exposure, with adaptive immunity still detectable at 2–6 months post-exposure [57]. In contrast, we have demonstrated that certain adaptive host transcriptional responses do occur early for both pathogens examined.

This study demonstrates the utility of using RNAseq with non-model organisms to identify loci that may be important in host responses to pathogens. Amphibians are a highly threatened group, faced with catastrophic declines driven in part by emerging infectious diseases. Whole genome data is currently only available for a single amphibian genus (*Xenopus*). As such, our transcriptome data for *R*. *temporaria* provides a valuable resource for another genus [58], much-needed insight into amphibian immunity, as well as information on specific responses to the two most important multi-host pathogens affecting amphibians. We were also able to identify candidate genes that could serve as markers for understanding the impacts of disease in wild populations and the adaptive potential of populations that are under threat. While this study has provided crucial insights into amphibian gene expression following exposure to pathogens, comparisons across life-history stages, time points post-exposure, and source populations with different previous infection statuses and genetic diversity will all be necessary to gain a more complete picture of the transcriptional responses to amphibian disease. We foresee this line of research offering exciting possibilities for the selection of individuals with disease resistance for captive breeding programmes for conservation.

## Supporting Information

S1 Text Re-allocating differentially expressed transcripts in the *Bd* vs. *Ranavirus* comparison. (DOCX)

S1 Table Sample RNA concentrations. (DOCX)

S2 Table CEGMA output summary; results of filtering assembly on CEG coverage. A) Full assembly, B) Filtered assembly on FPKM> = 1 for all replicates within at least one treatment.
(DOCX)

S1 Fig Expression pattern of re-allocated Bd vs. Ranavirus transcripts. Annotated transcripts from the *Bd* vs. *Ranavirus* (FDR<0.10) comparison that were also found in either *Bd* vs. control, *Ranavirus* vs. control or both prior to FDR filtering (protein name of best blast hit given).
(DOCX)

S1 File Full list of differentially transcribed genes (CSV)
