# Supplementary material for: A de novo Assembly of the Common Frog (Rana temporaria) Transcriptome and Comparison of Transcription Following Exposure to Ranavirus and Batrachochytrium dendrobatidis
Source: PLoS One. 2015 Jun 25;10(6):e0130500. doi: 10.1371/journal.pone.0130500 (PMC4481470; doi:10.1371/journal.pone.0130500)
Supplement: S1 Text — (DOCX) [file pone.0130500.s001.docx]

**S1 text. Reallocating differentially expressed transcripts in the *Bd* vs. *Ranavirus* comparison**

A unique set of differentially expressed transcripts was found in the *Bd* vs. *Ranavirus* comparison. 80 (25 annotated) of the total 136 transcripts in this comparison (FDR<0.10) were not found in either of the other comparisons at FDR<0.10. However the majority of the set were also present in one of the pathogen vs. control comparison sets before filtering on FDR (94 in *Bd* vs. control [45 with FDR>0.10]; 38 in *Ranavirus* vs. control [31 with FDR>0.10], 8 in both pathogen vs. controls, and 12 in neither pathogen vs. controls), i.e. they were differentially expressed but were not significant after filtering based on false discovery rate.

Greater variance among replicates in the control treatment in spite of similar mean expression levels relative to one of the pathogen treatments explains this pattern for many of these transcripts. For these transcripts that are differentially expressed in the *Bd* vs. *Ranavirus* comparison mean expression in the controls tends to be similar to one of the pathogens (pathogen A) but not the other (pathogen B). The greater variation in expression among samples in the control treatment masks the difference in expression compared to pathogen B that is seen in the comparison between pathogens (pathogen A effectively serves as a tighter ‘control’; see Supporting Information S4, which shows boxplots for the subset of annotated *Bd* vs. *Ranavirus* transcripts (FDR<0.10) that were allocated to one of the pathogen vs. control comparisons).
