# Supplementary material for: A de novo Assembly of the Common Frog (Rana temporaria) Transcriptome and Comparison of Transcription Following Exposure to Ranavirus and Batrachochytrium dendrobatidis
Source: PLoS One. 2015 Jun 25;10(6):e0130500. doi: 10.1371/journal.pone.0130500 (PMC4481470; doi:10.1371/journal.pone.0130500)
Supplement: S1 Table — (DOCX) [file pone.0130500.s002.docx]

S1 Table. Sample RNA concentrations.

| **Treatment** | **Pool ID** | **Number of individuals in pool** | **Pool Concentration (ng/µl)** |
| --- | --- | --- | --- |
| Control | CP1 | 5 | 319.95 |
|  | CP2 | 5 | 249.87 |
|  | CP3 | 5 | 251.35 |
| *Ranavirus* | RP1 | 5 | 248.70 |
|  | RP2 | 5 | 239.05 |
|  | RP3 | 5 | 191.55 |
| *Batrachochytrium dendrobatidis (Bd)* | BdP1 | 5 | 208.75 |
|  | BdP2 | 5 | 214.85 |
|  | BdP3 | 5 | 203.80 |
