# Supplementary material for: A de novo Assembly of the Common Frog (Rana temporaria) Transcriptome and Comparison of Transcription Following Exposure to Ranavirus and Batrachochytrium dendrobatidis
Source: PLoS One. 2015 Jun 25;10(6):e0130500. doi: 10.1371/journal.pone.0130500 (PMC4481470; doi:10.1371/journal.pone.0130500)
Supplement: S2 Table — A) Full assembly, B) Filtered assembly on FPKM> = 1 for all replicates within at least one treatment. (DOCX) [file pone.0130500.s003.docx]

S2 Table. CEGMA output summary; results of filtering assembly on CEG coverage. A) Full assembly, B) Filtered assembly on FPKM>=1 for all replicates within at least one treatment.
