# Supplementary material for: A de novo Assembly of the Common Frog (Rana temporaria) Transcriptome and Comparison of Transcription Following Exposure to Ranavirus and Batrachochytrium dendrobatidis
Source: PLoS One. 2015 Jun 25;10(6):e0130500. doi: 10.1371/journal.pone.0130500 (PMC4481470; doi:10.1371/journal.pone.0130500)
Supplement: S1 Fig — Annotated transcripts from the Bd vs. Ranavirus (FDR<0.10) comparison that were also found in either Bd vs. control, Ranavirus vs. control or both prior to FDR filtering (protein name of best blast hit given). (DOCX) [file pone.0130500.s004.docx]

**S1 Fig. Expression pattern of re-allocated Bd vs. Ranavirus transcripts.** Annotated transcripts from the *Bd* vs. *Ranavirus* (FDR<0.10) comparison that were also found in either *Bd* vs. control, *Ranavirus* vs. control or both prior to FDR filtering (protein name of best blast hit given).
